# Cranberry Polyphenol Extract (CPE) Oral Rinse Improves Salivary Microbiome in 6-n-Propylthiouracil (PROP) Non-Tasters and Palatability of Aronia Juice

**DOI:** 10.3390/ijms27093935

**Published:** 2026-04-28

**Authors:** Katrina Nguyen-DeMary, Sarah Vascellari, Mariano Mastinu, Melania Melis, Thomaz F. S. Bastiaanssen, Iole Tomassini Barbarossa, Beverly J. Tepper

**Affiliations:** 1 Department of Food Science and Center for Sensory Sciences & Innovation, Rutgers University, New Brunswick, NJ 08901-8520, USA; kn365@scarletmail.rutgers.edu; 2 Department of Biomedical Sciences, University of Cagliari, 09042 Monserrato, Italy; svascellari@unica.it (S.V.); mariano.mastinu@unica.it (M.M.); melaniamelis@unica.it (M.M.); tomassin@unica.it (I.T.B.); 3 Department of Psychiatry, Amsterdam University Medical Centers, 1105 AZ Amsterdam, The Netherlands; t.f.s.bastiaanssen@amsterdamumc.nl

**Keywords:** cranberry polyphenols, oral microbiome, PROP phenotype, astringency, oral biofilm

## Abstract

Sensitivity to the bitterness of 6-n-propylthiouracil (PROP) is controlled by variations in the *TAS2R38* gene. This phenotype is often used as a marker for individual differences in taste perception. Previous findings show that PROP taster status is associated with differences in the salivary microbiome. It is well known that diet and environmental factors influence the risk of oral disease, but there is far less evidence showing how genetic differences play a role. Forty-seven young, healthy, PROP taster-classified adults rinsed with a cranberry polyphenol extract (CPE) oral rinse (0.75 g/L CPE powder in spring water) twice daily for 11 days. Saliva was collected pre- and post-intervention for microbiome analysis using shotgun metagenomic sequencing. At the same time points, participants evaluated two astringent juices (cranberry and aronia berry) for key attributes. At baseline, PROP taster groups differed in their salivary microbiome compositions, but post-intervention, the groups had more similar bacterial compositions. Post-intervention, non-tasters showed decreases in the relative abundance of 15 bacterial species, including a significant reduction (*p* = 0.037) in *Eikenella corrodens*, which is one bacterium, among several others, involved in oral biofilm formation. Additionally, after the intervention, sourness was reduced, and overall liking increased significantly for aronia juice. Oral dysbiosis, a risk factor for oral disease, may be controlled by bactericidal mouthwashes. Our results suggest that CPE, a natural alternative to traditional bactericidal rinses, may selectively target pathobionts while preserving salivary microbiota diversity. CPE might also provide greater benefits to non-tasters, who are at greater risk for oral disease.

## 1. Introduction

Cranberries are rich in proanthocyanidins (PACs), which impart astringency to a variety of fruits (like grapes and other berries) and drinks (like tea, beer, and wine). The astringent, bitter, and sour qualities in these foods often act as barriers to consumption, given the evolutionary role that bitter taste has played in the avoidance of toxins from bitter plant foods [1]. Cranberries are unique because they contain PACs with A-type linkages, which are rarely found in other foods [2] and which have a wide variety of health benefits, including protection against pathogens, cardiovascular disease, certain cancers, and inflammatory diseases (Alzheimer’s, Parkinson’s, etc.) [3]. Cranberry juice has been used to prevent and treat urinary tract infections [4,5], though its effectiveness has recently been questioned [6]. Several studies have shown that cranberry polyphenols have potent anti-adhesion and anti-biofilm properties for bacteria in the urinary tract, specifically inhibiting the adhesion of *Escherichia coli* to the mucosa [7]. There is also evidence showing that cranberry polyphenols may act as anti-adhesion agents [7,8,9,10,11,12,13,14,15,16] and immunomodulators [17] in the oral cavity. Multiple exposure routes to polyphenols, such as rinsing [9,14,18], ingestion [15,19], and environmental exposure [20], have recently demonstrated promising effects in the oral environment.

The oral microbiome houses a wide diversity of microorganisms. The biofilm in the oral cavity is a complex ecosystem in which bacteria live in dynamic communities. In a healthy, eubiotic state, commensal microbes exist in harmony with their environment, contributing to the health of the host and homeostasis [21]. When a shift occurs in the environment turning favorable for pathobionts, dysbiosis occurs and the host becomes more susceptible to developing oral and even systemic diseases (i.e., dental caries, periodontitis, Alzheimer’s, diabetes mellitus, etc.). The transition from a healthy to a diseased oral state may be influenced by several factors, including environment (like diet, smoking, medications, oral hygiene practices, pH), host immune response, and genetic factors [22].

There are several recognized theories as to what causes oral disease. The Specific Pathogen Hypothesis suggests that specific bacteria in the oral cavity are periopathogenic and responsible for a range of oral diseases. This hypothesis led Socransky and colleagues to classify subgingival bacteria into six color complexes according to their association with the progression from oral health to oral disease [23,24,25]. The yellow and green complexes are comprised of bacteria typically associated with periodontal health in low abundance. These two complexes, together with the purple and blue complexes, represent the early colonizers that adhere to dental surfaces first, modulate the environment in the biofilm, and facilitate the attachment of other bacteria. Orange-complex bacteria typically appear in the biofilm after early colonizers; they attach to other bacteria that are adhered to dental surfaces and act as a bridge for early colonizers with late-colonizing bacteria. Lastly, the red complex represents late-colonizing periopathogens, which are found in high abundance in advanced stages of periodontitis [22,24].

Differences in taste sensitivity to synthetic bitter compounds, 6-n-propylthiouracil (PROP) and phenylthiocarbamide (PTC), have been the most studied taste phenotypes in humans [26] that may also play a role in food selection and dietary habits. Individuals who are highly sensitive to the taste of PROP are categorized as super-tasters (STs), while non-tasters (NTs) are taste-blind or have very low sensitivity to PROP, and medium-tasters (MTs) have moderate sensitivity [27]. Single-nucleotide polymorphisms in the *TAS2R38* gene, which encodes for the *TAS2R38* G-protein coupled receptor in taste cells, are responsible for the differing levels of bitter sensitivity [28]. In addition, there are reports that NTs have decreased sensitivity to sweetness [29], fat texture [30], and chemical irritants [31] compared to STs, who tend to have heightened perceptions of these qualities. Studies have found that PROP-sensitive individuals consume astringent berries less frequently than PROP-insensitive individuals [32], and they also perceive more bitterness, sourness, and astringency from high-tannin drinks (red wines) [33].

It has been repeatedly observed that NTs have higher rates of dental caries than STs [34,35,36], which might reflect their stronger preferences for sweet foods and higher intake of added sugars [37,38,39]. There is also mounting evidence that compared to NTs, STs have stronger innate immune responses that could promote greater dental caries protection [40,41,42]. These responses may be mediated by different families of salivary proteins, which are known to influence responses to astringency and impact oral health. For example, Melis and colleagues (2017) showed that PROP tasters had higher levels of acidic Proline-Rich Proteins (aPRP), basic Proline-Rich Proteins (bPRP), and Cystatins (Cysts) than NTs after stimulation with cranberry juice [43]. These differences suggest that PROP tasters may be able to mount and maintain higher levels of salivary proteins, especially those with antimicrobial properties like aPRPs and Cysts [44]. Finally, polymorphisms in the gustin (carbonic anhydrase 6) gene, which controls salivary pH homeostasis, are also associated with PROP sensitivity [45,46] and may represent another mechanism by which salivary composition may influence the oral milieu.

To date, only a few studies have investigated associations between PROP sensitivity and the salivary microbiota [18,47,48]; this relationship has largely been underexplored. Our previous study by Yousaf et al. (2022) was the first to examine the effects of cranberry polyphenols administered as a daily oral rinse on salivary microbiota [18]. Using the same cranberry polyphenol extract (CPE) rinsing protocol as used in the present study, Yousaf et al. (2022) showed that NTs and STs had different microbial compositions at baseline, but following the intervention, those differences disappeared [18]. This prior study used 16S rRNA sequencing, which limited our ability to identify species and strain differences that could provide insights for assessing oral disease risk. The present study used shotgun metagenomic sequencing to explore bacterial differences at the species and strain level, which was expected to provide a deeper understanding of microbial composition.

With this framework in mind, the present study was designed with three objectives. The first objective was to confirm differences in the salivary microbiome composition with respect to PROP phenotype at baseline. We hypothesized that at baseline, the oral microbiome composition of NTs would be consistent with greater oral disease risk as compared to STs. The second objective was to determine if daily rinsing with CPE oral rinse would alter the salivary microbiome composition, as we found in our prior study [18]. Socransky’s complexes, as mentioned above, were used as a descriptive ecological framework to examine ecological shifts, if any, in the salivary microbiome. We hypothesized that specific shifts in the bacterial composition following the intervention would confer greater benefits to NTs compared to STs, essentially providing more positive benefits to NTs. The third objective examined whether the intervention would alter perception and liking of cranberry juice and aronia berry juice (another high polyphenol astringent juice). Previous studies have shown that daily polyphenol ingestion altered the perception of astringency in both animals and humans [19,49]. However, no studies have examined whether the effects of oral rinsing with polyphenols, without ingestion, would have these same effects.

## 2. Results

### 2.1. Subject Characteristics

A breakdown of subject demographic characteristics is shown in Table 1. A total of 47 subjects participated in the study. Dietary intakes were assessed for differences among sex and PROP taster status cohorts (Table S1). Overall, dietary intake for males was higher than it was for females. There was a statistically significant difference in weight and intake of added sugars across sex, with males weighing more and consuming more added sugars on average. There were no differences in dietary intake between PROP taster status groups.

Salivary pH was measured before and after the intervention and assessed for differences. The mean pH of saliva was 7.2 at both pre- and post-intervention time points, and therefore, no overall change in pH was found after the intervention time. The mean pH for females at both time points was 7.1, while the mean pH for males was slightly higher at 7.3.

### 2.2. Salivary Microbiome in PROP Taster Groups

#### 2.2.1. Baseline Composition

**Figure 1:**
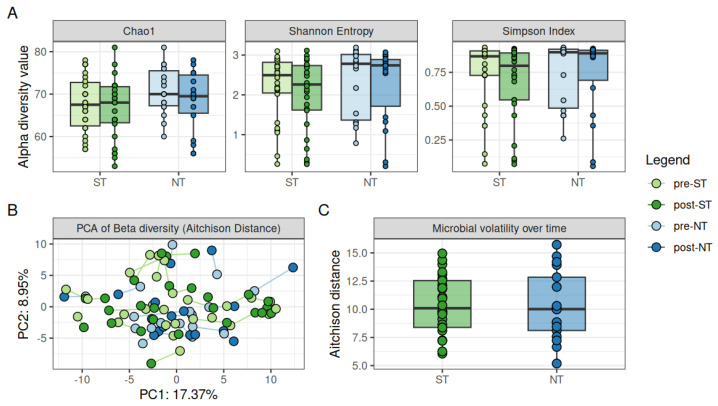
Alpha diversity, beta diversity, and volatility of ST and NT groups during the intervention times: (**A**) Alpha diversity of PROP taster status groups at the two time points shown by Chao1, Shannon, and Simpson indices; (**B**) Beta diversity is represented by PCA using Aitchison Distance; (**C**) Microbial volatility is calculated by the Aitchison distance between pre- and post-intervention. Statistical significance is set at *p* < 0.05. NT = non-taster (*n* = 18), ST = super-taster (*n* = 26).

**Figure 2:**
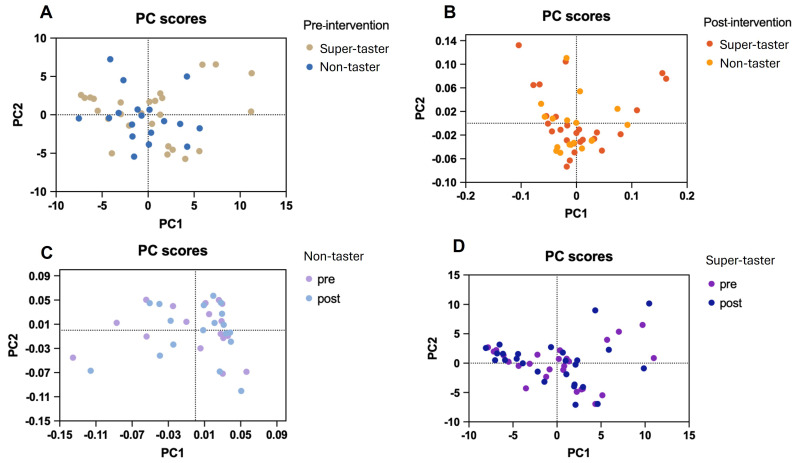
PCA plots show a comparison of PROP taster groups at each intervention time and an intervention time comparison for each taster group (*n* = 44): (**A**) Pre-intervention; (**B**) Post-intervention; (**C**) Non-taster; and (**D**) Super-taster.

The salivary microbiome composition was assessed for PROP taster status groups at baseline. No differences were identified for beta and alpha diversity between ST and NT groups, as measured by evenness and richness using Chao1, Shannon, and Simpson indices (Figure 1). The Chao1 index is an estimate of species richness that accounts for low-abundance species, while the Shannon index incorporates both richness and evenness, and the Simpson index encompasses richness and abundance. To assess individual bacterial species, a cutoff criterion of an increase or decrease in more than 50% of subjects was chosen, which led to the identification of 124 bacterial species overall. Differential abundance analysis indicated a pattern of higher relative abundance of 8 bacterial species and lower relative abundance of 1 species in NTs compared with STs (Table 2). Species that were greater for NTs than STs in relative abundance were *Prevotella nigrescens*, *Oribacterium sinus*, *Selenomonas_u_s*, *Selenomonas infelix*, *Campylobacter_u_s*, *Campylobacter concisus*, *Eikenella corrodens*, and *Eikenella halliae.* The one species that was lower in NTs than STs in relative abundance was *Peptinophilus lacrimalis*. After multiple comparison correction, the differences in bacterial species were not significant. Overall, for the top 124 bacterial species, PROP taster status groups showed a slight variation in salivary microbiome composition at baseline, whereas no clear separation was observed after the intervention (Figure 2). No differences were observed with ST or NT groups in bacterial composition from pre- to post-intervention.

#### 2.2.2. Changes as a Function of the Intervention

Microbial community structure was evaluated by analyzing alpha diversity (Shannon, Chao1 and Simpson indices), beta diversity, and volatility across groups over time. Although a slight trend toward higher alpha diversity was observed at baseline in both NT and ST groups, this difference was not statistically significant and likely reflects natural interindividual variability rather than a consistent difference over time. No significant differences in beta diversity were observed between pre- and post-intervention time points in either ST or NT groups. Additionally, microbial volatility did not significantly change in PROP taster status groups as an effect of the intervention (Figure 1).

With regard to within-subjects differences in PROP taster status groups and intervention time, bacterial species that were significantly different before and after multiple comparisons correction tests are shown in Table 3. After the intervention, an overall decrease was observed in the relative abundance of bacteria for NTs. Fifteen bacteria decreased in relative abundance for NTs, including *Actinobaculum_u_s*, *Actinomyces gerencseriae*, *Actinomyces massiliensis*, *Corynebacterium matruchotii*, *Capnocytophaga granulosa*, *Capnocytophaga ochracea*, *Prevotella nigrescens*, *Prevotella oris*, *Oribacterium_u_s*, *Oribacterium asaccharolyticum*, *Selenomonas infelix*, *Streptococcus sanguinis*, *Campylobacter rectus*, *Eikenella corrodens*, and *Kingella denitrificans.* After multiple comparison correction was applied, *E. corrodens* remained significantly reduced (*p* = 0.037) in relative abundance as an effect of the intervention. For STs, there were increases in 10 bacteria after the intervention (*Atopobium_u_s*, *Prevotella nigrescens*, *Prevotella scopos*, *Lachnoanaerobaculum orale*, *Lachnospiraceae_u_s*, *Streptococcus vestibularis*, *Veillonella_u_s*, *Campylobacter_u_s*, *Campylobacter concisus*, and *Aggregatibacter aphrophilus*) and decreases in 5 bacteria (*Rothia aeria*, *Streptococcus xiaochunlingii*, *Streptococcus viridans*, *Lautropia mirabilis*, and *Neisseria polysaccharea*). After multiple comparisons correction, the differences for STs did not show statistical significance.

#### 2.2.3. Changes in Socransky’s Complex Bacteria in PROP Taster Groups

**Figure 3:**
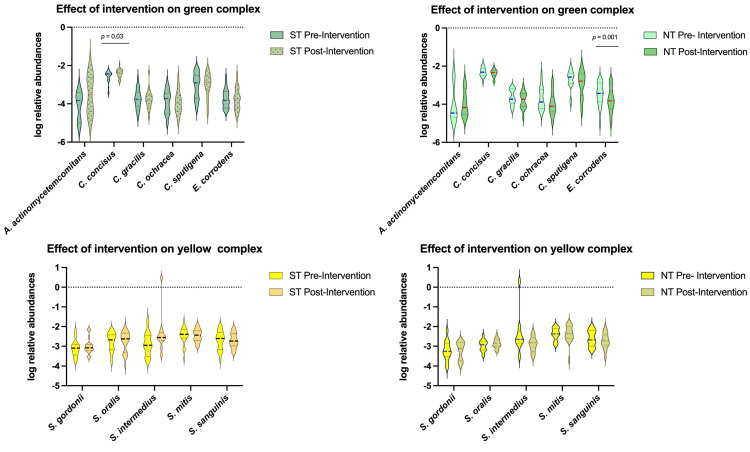

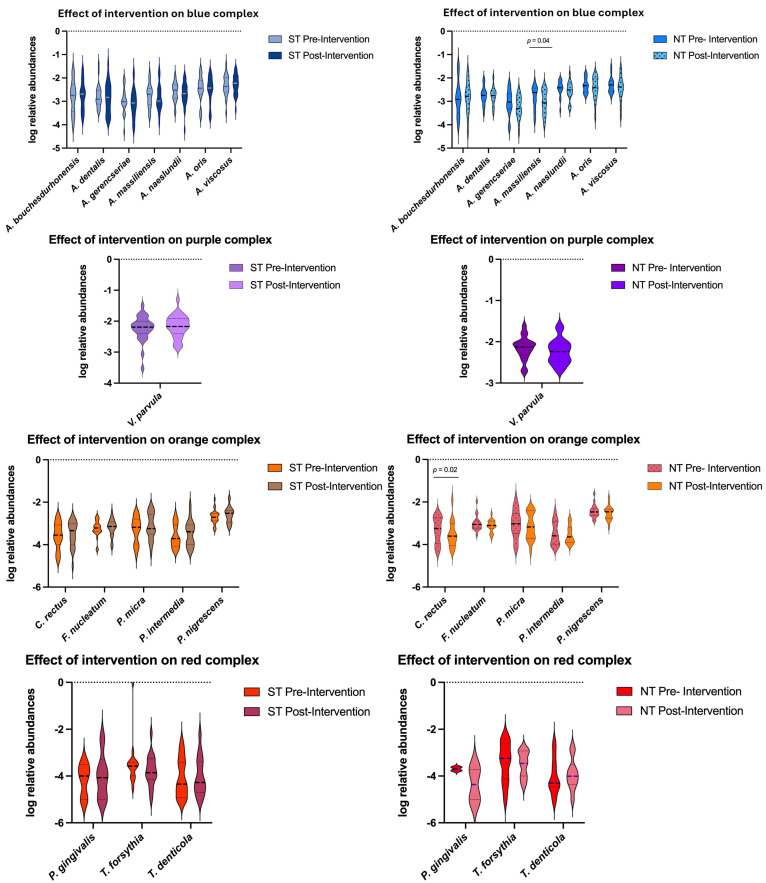
Violin plots showing the log relative abundance of bacteria in Socransky’s color-coded complexes after the intervention time for PROP taster status groups. Multiple Wilcoxon signed rank test was performed, followed by the Holm–Sidak correction test. The color of each plot corresponds to the Socransky’s color-coded complex that the bacteria belong in: green, yellow, blue, purple, orange, and red. Statistical significance is set at *p* < 0.05. NT = non-taster (*n* = 18), ST = super-taster (*n* = 26).

Next, bacteria in Socransky’s complexes were examined for changes in relative abundance for ST and NT groups as an effect of the intervention. Socransky’s complexes classify bacteria into 6 color-coded groups based on their involvement in the initiation and progression of periodontal disease. The main effects of the intervention were observed in the NT group, which showed an overall decreasing trend in the relative abundance of bacteria recognized in Socransky’s complexes (Figure 3). The significant changes concerned both early colonizers belonging to the green and blue complexes and secondary colonizers belonging to the orange complex. The relative abundances of primary colonizers such as *E. corrodens* (*p* = 0.001) within the green complex and *Actinomyces massiliensis* (*p* = 0.04) within the blue complex were significantly reduced after the intervention. For the orange complex, there was a significant reduction in the relative abundance of secondary colonizer *Campylobacter rectus* (*p* = 0.02) for the NT group. There were no statistically significant changes found for the yellow, purple, and red complexes. Conversely, the CPE intervention elicited a more limited and opposite effect on primary colonizers in the ST group. The only change found concerned a significant increase in the relative abundance of Campylobacter concisus (*p* = 0.03) within the green complex.

PROP taster status groups were compared for the relative abundance of Socransky’s complex bacteria at baseline and post-intervention measures. *Prevotella nigrescens* of the orange complex was significantly higher (*p* = 0.02) in relative abundance for NTs compared to STs at baseline (Figure S1). There were no other statistically significant differences between the groups at baseline or post-intervention.

### 2.3. Overall Effects of the Intervention in the Salivary Microbiome—All Participants

Bacterial species that had significantly changed across all subjects, comparing baseline to post-intervention, before and after multiple comparisons correction tests, are shown in Table 4. Following the intervention, there were 7 increases and 6 decreases in the relative abundance of bacterial species. Increases were seen in *Atopobium_u_s*, *Prevotella scopos*, *TM7 phylum sp. oral taxon 3*, *Lachnoanaerobaculum_u_s*, *Streptococcus thermophilus*, *Streptococcus vestibularis*, *and Veillonella_u_s*. On the other hand, decreases were observed in *Capnocytophaga ochracea*, *Lautropia mirabilis*, *Lautropia dentalis*, *Kingella denitrificans*, *Neisseria cinerea*, *and Neisseria polysaccharea* before multiple comparisons correction. Once multiple comparisons correction was applied, *S. vestibularis* remained significantly higher in relative abundance after the intervention (*p* = 0.039), but no other significant differences in relative abundance were observed.

### 2.4. Sensory Evaluation

#### 2.4.1. Overall Group Differences

**Figure 4:**
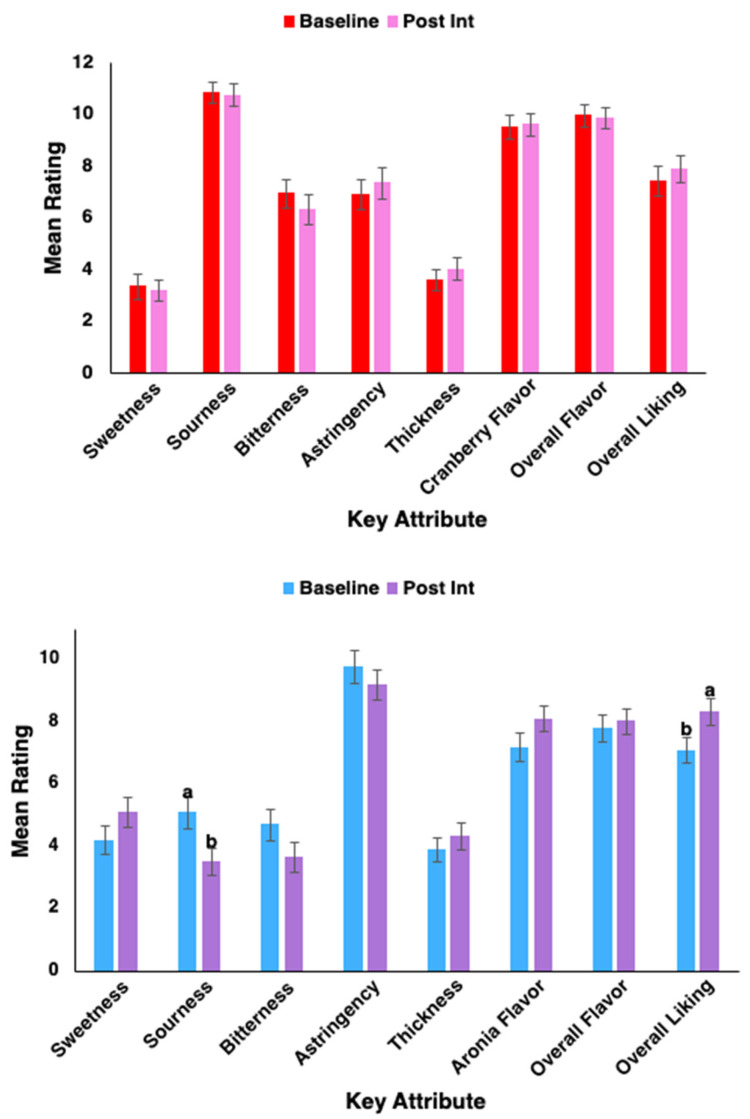
Mean intensity ratings for sensory evaluation of key attributes for cranberry (**top**) and aronia berry (**bottom**) juices after the intervention period. Error bars represent the standard error of the mean (SEM). Repeated measures MANOVA was used to detect statistical significance at *p* < 0.05. Different letters (a,b) represent significant differences (*n* = 47).

Ratings for astringent juices were assessed for statistically significant differences over sex, PROP taster status, and intervention time. This section will cover differences found for the overall group as an effect of the intervention. For aronia berry juice, sourness was perceived as significantly reduced (*p* = 0.019) after the intervention, and overall liking was significantly increased (*p* = 0.037) (Figure 4). Though not statistically significant, trends for decreasing bitterness and astringency were observed, while there was an increasing trend for aronia flavor. For cranberry juice, there were no significant differences found between intervention times; trends for increasing astringency and overall liking, along with decreasing bitterness, were observed post-intervention.

#### 2.4.2. Sex and PROP Taster Status Effects

Astringent juice ratings were assessed for differences between PROP taster status groups and sex. There were some trends observed for PROP taster status groups after the intervention time, but there were no statistically significant differences (Table S2, Figure S2). In addition, there was a statistically significant crossover interaction (*F*_1,90_ = 4.81, *p* = 0.031) between the effects of intervention time and sex on overall flavor ratings for aronia berry juice. The overall flavor mean rating for males increased after the intervention, while the mean rating for females decreased. There were no statistically significant differences for astringency sensations with the Rate-All-That-Apply (RATA) (Table S3).

#### 2.4.3. Correlation Between Bacteria and Sensory Perception

Next, correlations between bacteria and juice sensory ratings were explored for PROP taster groups and intervention time. A positive correlation was found, using Spearman’s rank correlation test, for the percent relative abundance of *E. corrodens* and astringency rating of cranberry juice post-intervention (*r* = 0.514, *p* = 0.029). However, as no correction for multiple testing was applied at this step, this finding should be considered preliminary. There were no other significant correlations between bacteria and sensory ratings for cranberry and aronia berry juices in PROP taster groups.

## 3. Discussion

### 3.1. Baseline Salivary Microbiome

#### 3.1.1. PROP Taster Groups

The first objective of the study was to assess whether there was baseline variation in the salivary microbiome composition from unstimulated saliva samples associated with PROP sensitivity. Although microbiome composition showed trend-level variation between NT and ST groups at baseline (Table 2), none of these differences remained statistically significant after multiple testing correction. The present findings differ from our previous study [18], which reported higher relative abundance in several bacterial genera in the saliva of STs compared to NTs, as well as differences in alpha and beta diversity between the groups, which we did not observe in the present study. Cattaneo et al. (2019) reported a higher relative abundance of several bacterial genera in STs relative to NTs, but the identified genera were different across the two studies (besides *Actinomyces*) [47]. This lack of consistency across studies may reflect variability in demographic characteristics such as ethnicity and lifestyle across the relatively small sample sizes in these studies. Specifically, the present study investigated a larger sample size (*n* = 47) than the study by Yousaf et al. (2022; *n* = 20), but the ethnic diversities in the two studies were different [18]. The study by Cattaneo et al. (2019; *n* = 59) was conducted in Italy in a University community, but the subjects were not further described [47]. Besides, Cattaneo et al. (2019) collected tongue swabs for DNA analysis rather than whole mouth saliva, as we did here, which could also lead to differences in study outcomes [47]. Thus, the first research query of this study, whether the unstimulated oral microbiome differs between NTs and STs, remains an unanswered question. This issue requires further investigation. 

#### 3.1.2. Socransky’s Complexes

The baseline salivary microbiome in PROP taster status groups was further assessed for the bacteria in Socransky’s complexes, which represent bacteria involved in the progression of periodontal disease. *P. nigrescens*, from the orange complex, was significantly greater in abundance than NTs at baseline compared to STs, whereas there were no differences observed for other complexes (Figure S1). This species is an oral pathogen frequently detected in the subgingival plaque of individuals with periodontitis [50,51]. Following the CPE intervention, the differences between PROP taster groups in *P. nigrescens* abundance disappeared. This suggests that the CPE intervention may have shifted the salivary microbiome of NTs to a profile more closely resembling STs, one that reflects a state of good oral health.

### 3.2. Pre- and Post-Intervention Salivary Microbiome Comparisons

#### 3.2.1. PROP Taster Status

The present study revealed no differences in bacterial composition after the intervention in ST or NT groups when examined separately in PCAs. Beta diversity was also not different between groups after the intervention, and microbial volatility did not change after the intervention period for PROP taster status groups (Figure 1). Altogether, these results suggest that CPE intervention did not impact the overall oral microbial community and ecosystem stability in participants. This outcome is not surprising, as stability to changes in the salivary microbiome environment over a short period of time is expected in healthy individuals. This contrasts with the higher degree of volatility observed in high stress [52] or disease states [53].

As mentioned previously, after multiple comparison correction was applied, only *E. corrodens* was reduced in NTs as a result of the intervention. *E. corrodens* is a Gram-negative facultative anaerobe, with its primary ecological niche in the oral cavity being dental plaque. *E. corrodens* is a bacterium implicated, among others, in the development of oral disease. For example, there are several virulence factors associated with *E. corrodens*, including but not limited to adhesins, lipopolysaccharide [54], and pili [55], which are characteristics that facilitate oral surface attachment and immune response activation, thus promoting biofilm formation and disease progression. It is a native microorganism of the oral cavity that may be an opportunistic pathogen, playing a role in the progression of periodontitis in young subjects [56]. *E. corrodens* is associated with the formation of the biofilm in initial stages, enabling the bridging of early colonizers with late colonizers [57,58]. Notably, *E. corrodens* appears in higher frequencies in juvenile and adult periodontitis subjects compared to healthy subjects [59]. This finding suggests that when PROP taster status groups are looked at separately, the CPE oral rinse significantly reduces the relative abundance of a bacterium in NTs associated with disease in the oral cavity.

#### 3.2.2. Socransky’s Complexes

The effect of the CPE oral rinse on the salivary microbiome in PROP taster status groups was further evaluated by examining taxa classified within Socransky’s complexes, originally described based on subgingival plaque in relation to periodontal disease progression. Although saliva provides a composite representation of the oral microbiome by integrating microorganisms from multiple oral niches, it does not specifically reflect the subgingival microbiota. Accordingly, as all participants were periodontally healthy, this classification was used solely as a descriptive framework to explore potential ecological shifts in the salivary microbiome following the intervention, rather than a periodontal-specific effect.

Overall, decreasing trends were observed in NTs for these bacteria, but STs showed both increases and decreases (Figure 3). Statistically significant changes were observed in the green, blue, and orange complexes. No statistically significant changes were found for the red complex, which was expected since these bacteria are typically present in low abundance, or not present, in individuals with good oral health.

In NTs, *E. corrodens* belonging to the green complex was significantly reduced as an effect of the intervention, along with *A. massiliensis* from the blue complex and *C. rectus* from the orange complex. *E. corrodens* was described previously as being involved in the initiation and progression of periodontitis. *A. massiliensis* was detected in higher percentages in the supragingival biofilm of chronic periodontitis patients compared to healthy individuals [60], while *C. rectus* has frequently been isolated from adult and juvenile periodontitis patients and less frequently isolated from individuals with good oral health [61]. For STs, *C. concisus* from the green complex was significantly increased. *C. concisus* has been isolated from individuals with gingivitis and periodontitis and is known to be associated with these conditions [62].

It is important to note that there were several statistically significant reductions in bacteria involved in the initiation and progression of periodontal disease in NTs, compared to STs, where this was not observed. Also, NTs were found to have an overall decreasing trend in bacteria associated with periodontally diseased states, whereas STs were shown to have both increases and decreases, suggesting that the CPE oral rinse intervention had a greater benefit in NT-classified individuals. As mentioned previously, NTs are thought to be at higher risk for oral dysbiosis compared to STs [40,41,42]. That being said, cranberry polyphenols may have selective action in the oral cavity, potentially being more effective in individuals with higher oral disease risk, while having a smaller, less potent effect in individuals with greater protection against oral disease. This enables the possibility of using cranberry polyphenols in personalized nutrition, specifically targeting individuals with greater oral disease risk based on their genes. These results show that the CPE oral rinse acted to inhibit oral disease-associated bacteria in NTs, which in turn modulated the microbial community to a state that reflects good oral health and reduced oral disease risk. The action by which cranberry polyphenols exert these benefits on the salivary microbiome may be through attachment to hydrophobic cell surface proteins on pathogenic bacteria, thus hindering adhesion to the tooth surface or exopolysaccharides present in the dental biofilm [7,14], by binding to and inhibiting proteins like glucosyltransferases (GTFs) that catalyze the development of the biofilm [8], or by inhibiting quorum sensing of pathogenic bacteria [63].

Broad-spectrum antiseptics (e.g., chlorhexidine) have traditionally been used as antimicrobial agents in oral care, but they can have a double effect. They kill both commensals and pathogens, shifting toward an acidic environment favorable for cariogenic bacteria, which can disrupt microbial homeostasis and contribute to antimicrobial resistance [64,65]. Anti-adhesion agents, including cranberries [9,13], have been investigated as an alternative to standard treatment. The goal for anti-adhesion agents is to inhibit the adhesion of bacteria to surfaces in the oral cavity, thereby inhibiting biofilm formation and modulating the oral microbiome without affecting the viability of the microbes [9,13]. This allows for plaque control by reducing the ability of pathogens to cause disease, thus acting as an important long-term method for controlling dental caries and periodontal disease and improving microbiome dysbiosis [66].

#### 3.2.3. All Participants

Differential relative abundance analysis of bacteria performed for all participants showed increases in 7 and decreases in 6 bacterial species after the intervention (Table 4). One species, *S. vestibularis*, was identified as significantly increased in participants as a result of the CPE oral rinse intervention after multiple comparison correction. *S. vestibularis* is a Gram-positive facultative anaerobe that has been mainly isolated from the vestibular mucosa of the oral cavity. One identifying characteristic of *S. vestibularis* is that it cannot convert sucrose into exopolysaccharides, which suggests that it likely would not contribute to the formation or maturation of the oral biofilm [67]. Additionally, this bacterial species produces hydrogen peroxide and urease, by which ammonia is then produced, ultimately increasing the local pH [68]. A pH increase in the oral environment would likely hinder the growth of aciduric and acidogenic bacteria, which are associated with caries development, ultimately leading to a shift in the microbial community to one associated with health. In subjects with periodontitis, *S. vestibularis* was the most abundant species in healthy gingival tissue sites but was found to be significantly lower in abundance for diseased sites [69]. This suggests that the CPE oral rinse had a beneficial effect overall in the microbiome of all subjects, significantly increasing a bacterium associated with health.

Additionally, the most abundant genera of the baseline salivary microbiome composition in all subjects consisted of *Streptococcus*, *Prevotella*, and *Veillonella*. These findings are in line with the dominant genera found in other studies that used unstimulated saliva [70,71].

### 3.3. Changes in Sensory Ratings and pH Post-Intervention

Astringency perception did not change between the groups or for all subjects after the intervention with the cranberry juice. This same outcome was also observed in our previous study [18]. However, a decrease in sourness and a reciprocal increase in overall liking were observed for the aronia berry juice at the end of the intervention for all subjects (Figure 4). Examination of the data by PROP taster groups showed that these changes were presumably driven by STs (Table S2, Figure S2), although the PROP-related effects were not statistically significant. Why twice-daily rinsing with CPE altered responses to aronia berry juice but not cranberry juice, especially for STs, is unclear, but several possible explanations deserve attention. First, visual examination of the sensory ratings for all subjects (Figure 4) reveals that cranberry juice provoked more sourness and less astringency than aronia berry juice, which showed the opposite profile. The more astringent aronia berry juice might simply have invoked greater changes in sensory responses following the intervention than the less astringent cranberry juice. Second, previous studies have shown that dietary exposure to polyphenols reduces taste aversion to tannic acid in rodents [49] and modulates taste responses to phenolic-rich foods in humans [19]. These changes are thought to be mediated by upregulation of salivary proteins that bind polyphenols, reducing the perception of astringency [49]. Our previous studies with acute oral stimulation of cranberry polyphenols show that STs released more of these proteins [72]. Thus, it is possible that STs are able to mount a more robust salivary protein response, leading to a greater reduction in aversive responses to aronia berry juice.

Why we observed decreased sourness perception but not astringency perception is also unclear. Subjects may have conflated sourness or bitterness with astringency, as this is commonly seen with panelists who are not highly trained [73]. A familiarization session was included before the evaluations began, but it’s possible that the subjects needed more time and training to better differentiate these qualities.

Finally, it is important to note that the present study achieved changes in liking with twice-daily rinsing with CPE in contrast to previous studies, which utilized daily polyphenol ingestion [19,49]. These findings suggest that merely rinsing with CPE daily, without ingestion, may be sufficient for reducing taste aversions to polyphenols and may confer some positive benefits to the oral microbiome, at least for some individuals.

After the intervention, the relative abundance of *E. corrodens* was positively correlated with astringency ratings in cranberry juice for NTs, but not in STs. That is, perceived astringency was higher in NTs with a greater relative abundance of *E. corrodens* in saliva. This was an unanticipated finding that we speculate may be related to salivary protein changes. Future work should investigate this relationship.

Our findings for salivary pH are consistent with these values reported in the literature. The typical pH of human saliva is 6.75–7.25 [74], with slightly lower values for females relative to males [75]. It is important to note that salivary pH remained stable after the intervention. Daily rinsing with CPE may have indirectly contributed to the stability of oral pH in our participants through its antimicrobial and anti-inflammatory effects and by inhibiting acid-producing bacteria. These effects contribute to a stable oral environment and prevent disease [76]. We used pH test strips, which provide only a rough estimate of saliva pH. It is possible that there were subtle shifts in pH after the intervention period that we were unable to measure. In the future, more precise pH measurement may be needed to reveal small changes, if they occur.

### 3.4. Limitations

There are several limitations of the present study that are worth discussing. First, we tested 47 subjects, which is a larger population size and a more diverse subject pool than our previous study. In the future, it would be beneficial to test a larger number of subjects to increase statistical power and validate the results. In addition, the intervention period in this study was 11 days long to maintain consistency with our previous study. Future studies should consider implementing a longer intervention period and/or rebound period to understand if more substantial changes occur with more exposure, and if ecological shifts in the salivary microbiome remain after stopping the intervention. Moreover, we tested young, healthy, mainly college-educated individuals, with the majority being Caucasian (although our population comprised different ethnicities). Future studies should account for any differential disease risks that may exist in marginalized groups. Lastly, studying the effects of the intervention in individuals with gingivitis or periodontal disease in the future would be an area of interest.

## 4. Materials and Methods

### 4.1. Subject Recruitment and Screening

Individuals were recruited via email distribution lists and flyers posted at Rutgers University, New Brunswick campus. Interested individuals filled out a prescreening survey via Qualtrics, then responses were reviewed to assess eligibility based on inclusion and exclusion criteria. Those who qualified were then contacted to participate in a screening session.

Individuals were 18–50 years old, non-smoking, weight stable (not more than ± 5 lb. weight change in the last 6 months), familiar with cranberry juice, not pregnant or lactating, free from metabolic diseases, psychiatric illnesses, oral health problems, and not taking any medications that alter taste/smell function, microbiota, or food intake. Those with sensitivities, allergies, or restrictions to foods and food aromas used in the study were excluded. Individuals who had taste/smell dysfunction, oral piercings, were not current on routine checkups by an oral/dental health professional, or had not undergone dental cleaning by an oral/dental health professional in the last year were excluded. To participate in the study, individuals must agree to follow the intervention schedule and use the materials as instructed, along with refraining from consuming a list of foods and other oral rinse products during the duration of their participation in the study.

In addition, recruitment was based on PROP taster status, with NTs and STs included and MTs excluded. The methods used to classify PROP taster status are described in the following paragraph.

Eligible individuals were invited to participate in an in-person screening session. During the screening session, an informed consent document was provided to volunteers. Next, volunteers filled out Demographic and Oral Health Questionnaires to collect information about general demographic data, age, sex, height, weight, oral health and hygiene habits, and medications that might impact taste or smell function or microbiota. After this, the ‘Sniffin’ Sticks 12-Identification Test’ (St. Croix Sensory Inc., Stillwater, MN, USA) was performed with 3 pre-selected sticks to assess normal smell ability [77]. Those who got less than two odors correct did not qualify. Following this test, volunteers were screened for PROP sensitivity. Classifying individuals into ST, MT, and NT groups included the following steps: individuals placed a filter paper disk on the tip of their tongue impregnated with 1.0 mol/L NaCl until wet, then rated the taste intensity using a 100 mm end anchored labeled magnitude scale (LMS; 0 = Barely Detectable, 100 = Strongest Imaginable). The same procedure was followed with a second paper disk impregnated with 50 mmol/L PROP. Individuals were instructed to drink spring water before and in between samples. Individuals were then categorized into 3 groups based on their ratings of the PROP disk: those that rate ≤ 15 mm are considered NTs, > 67 mm are considered as STs, and in between are considered MTs [78]. PROP MTs were not included in the study.

The study was conducted at the Sensory Evaluation Lab at Rutgers University, New Brunswick, a standard sensory environment with booths. The study was approved by Rutgers University’s electronic Institutional Review Board (Protocol ID: Pro2022000271) and registered with ClinicalTrials.gov PRS (ClinicalTrials.gov ID: NCT04107688). Written informed consent was obtained from all participants, and participants were monetarily compensated for their time.

### 4.2. Oral Rinse and Juice Preparation

Oral rinse samples were prepared using cranberry polyphenol extract (CPE) powder (Ocean Spray Inc., Lakeville-Middleboro, MA, USA) combined with spring water (0.75 g/L). The CPE powder contains 53.5% high molecular-weight proanthocyanidins. Spring water was used for the control sample. Samples were poured into amber glass vials (30 mL per vial) and refrigerated at 4 °C until distributed to subjects.

Cranberry juice and aronia berry juice were prepared separately using the same procedure. Frozen cranberries (Cape Cod Select, Carver, MA, USA) were purchased from a local grocery store. Frozen aronia berries (Mae’s Health and Wellness, Omaha, NE, USA) were ordered online. Berries were stored in a −20 °C freezer. In a saucepan, 300 g of either berry and 648 g of spring water were added, then brought to a boil on the stovetop. After reaching a boil, the pan was loosely covered and reduced to medium heat for 10 min. Next, the juice was strained through a colander lined with a cheesecloth, then brought to room temperature. Taste solutions evaluated in the first session included sour (1.0 g/L citric acid), bitter (0.6 g/L caffeine), and astringent (1.38 g/L alum) solutions. All juices and solutions were stored in the refrigerator at 4 °C and brought to room temperature for subject evaluation.

### 4.3. Saliva Collection and Analysis

At the beginning of the sessions, subjects provided a small sample of saliva for DNA and microbiome analysis. Saliva was collected in the morning between 9 a.m. and 11 a.m. to limit variation due to circadian rhythm [79], and subjects fasted overnight before each session. The salivary pH for each subject was recorded for all sessions using pH test strips (4.5–10.0, VWR). During Session 1, 1 mL of untreated saliva for each subject was kept at −80 °C for DNA analysis. For Sessions 2 and 3, subjects provided saliva samples that were transferred to three microcentrifuge tubes kept cold on ice and then stored at −80 °C.

To have a holistic representation of microbiota composition in the oral cavity, a total of 47 saliva samples were collected for shotgun metagenomic sequencing. However, one sample was not analyzed due to a procedural error in processing the sample. Saliva samples were delivered to Cosmos ID Inc. in Germantown, MD, on dry ice for shotgun metagenomic sequencing and bioinformatics analysis. DNA was extracted and quantified using Qubit Flex fluorometer and Qubit™ dsDNA HS Assay Kit (Thermofisher Scientific, Waltham, MA, USA). DNA libraries were prepared using the xGen DNA Library Prep Kit (IDT) and xGen Normalase UDI Primers with a total DNA input of 1.5 ng. Genomic DNA was fragmented using a proportional amount of IDT xGEN fragmentation enzyme. Unique dual indexes were added to each sample, followed by 10 cycles of PCR to construct libraries. DNA libraries were purified using AMpure magnetic Beads (Beckman Coulter, Brea, CA, USA) and eluted in QIAGEN EB buffer (QIAGEN, Hilden, Germany). Qubit fluorometer and Qubit™ dsDNA Assay Kit were used to quantify DNA libraries. The Illumina NovaSeq X Plus platform was used to sequence libraries at 2x150bp. Bioinformatics analysis was performed using a data-mining k-mer algorithm that groups millions of short sequence reads into distinct genomes. Two separate comparators were utilized. The first was a pre-computation phase for reference databases, where the input is databases of reference genomes, virulence markers, and antimicrobial resistance markers, and the output is a phylogeny tree of microbes with sets of variable-length k-mer fingerprints that are uniquely associated with certain branches and leaves of the tree. The second was a per-sample computational phase that searched the hundreds of millions of short sequencing reads from draft de novo assemblies against the fingerprint sets and GenBook database, allowing for detection and taxonomic classification of microbial next-generation sequencing reads. The statistics were analyzed to gather detailed taxonomic classification and relative abundance. To exclude false positive identifications, the results were filtered using a filtering threshold derived based on internal statistical scores that were determined by analyzing several diverse metagenomes.

### 4.4. Sensory Evaluation of Juice Samples

Participants were provided samples for sensory evaluation for all three sessions. During Session 1, participants were provided with several taste samples as a familiarization exercise to help them differentiate several key attributes in complex polyphenol-rich beverages. They rated sour, bitter, and astringent solutions on 15-point end-anchored intensity scales (1 = Very Weak, 15 = Very Strong). Since astringency is not a commonly used term, the following definition was provided to subjects: a group of sensations involving drying, roughing, and puckering sensations in the mouth. Subjects were also given definitions for each astringency sensation, then they were asked to rate-all-that-apply (RATA) on the same 15-point end-anchored intensity scales. Lastly, the subjects were provided with a sample of cranberry juice. They were asked to rate the sample for several key attributes (sweetness, sourness, bitterness, astringency, thickness, cranberry flavor, overall flavor) on 15-point end-anchored intensity scales (1 = Very Weak, 15 = Very Strong), RATA for the astringency sensations, and overall liking (1 = Dislike Extremely, 7.5 = Neither Like nor Dislike, 15 = Like Extremely). Sessions 2 and 3 were identical, where subjects were served two juice samples, cranberry and aronia berry juices, and evaluated these samples for the same key attributes as the cranberry juice sample from Session 1, along with RATA for astringency sensations, and overall liking, with the aronia berry juice sample being evaluated for “aronia flavor” instead of “cranberry flavor.” The data from these two sessions were used to determine the effect of the CPE oral rinse on sensory perception. Subjects were instructed to drink spring water before evaluating and between samples. All samples were portioned into clear soufflé cups (15 mL) and served separately at room temperature. Subjects evaluated samples in individual booths.

### 4.5. Intervention Protocol

**Figure 5:**
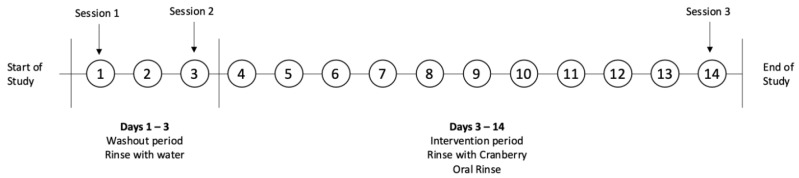
Timeline of the 14-day study, including 3 sessions of subject testing, a washout period, and an intervention period.

The timeline of the study is described in Figure 5. Qualified subjects participated in a 2-week clinical trial. The first part of the study included a washout period, where subjects rinsed their mouths with spring water (control) from day 1 to day 3. Following this, subjects rinsed with CPE oral rinse from day 4 to day 14. Subjects were instructed to follow their daily oral routine and to rinse their mouth twice a day after brushing their teeth. They were asked to rinse with the samples at relatively the same time each day, morning and evening. On testing days, subjects were asked to use the rinse 1 h before their session time. Subjects were asked to avoid using other oral rinse products during the 2-week participation period, along with avoiding consumption of A-type PAC-rich foods (cranberries and their derivatives, plums, avocados, peanuts, cinnamon, and curry powder). The same procedure was followed for the washout and intervention periods. On day 1, subjects provided saliva for DNA analysis, then were provided with taste solutions and cranberry juice as a familiarization task. A food frequency questionnaire was used to collect information about dietary intakes over the past month through questions about 135 foods and beverages and 26 dietary supplements [80]. This was included in the study to understand the typical carbohydrate and sugar intake of the participants, since these factors are known to influence the oral microbiome composition. On days 3 and 14, subjects provided saliva for salivary microbiome analysis, then were asked to evaluate samples of cranberry and aronia berry juices for key sensory attributes.

### 4.6. Statistical Analyses

Alpha diversity was obtained by the first three Hill numbers, including Chao1, Simpson, and Shannon indices, by genus-level differentiation. Principal component analysis (PCA) of beta diversity and volatility was measured as the distance between pre- and post-intervention and calculated using Aitchison distance between the two time points. Mann–Whitney U test or Multiple Wilcoxon signed rank test, followed by the Holm–Sidak method correction test for multiple comparisons, were performed to evaluate the significant differences in bacterial relative abundance between taster groups at baseline and pre- vs. post-intervention. Multivariate PCA was performed to evaluate data patterns and highlight salivary microbiome composition similarities and differences between different groups (PROP taster groups and intervention time) and observe clusters of data points. In the dietary intake analysis, two outliers were identified for carbohydrate, total, and added sugars intake based on assessment of standardized residuals that were outside of the range [−1.96, 1.96]. These outliers were then excluded from the microbiome and dietary intake analyses due to the strong effect that sugars and carbohydrates have in promoting the growth of aciduric cariogenic bacteria [81,82]. All statistical analyses for the salivary microbiome data were plotted using the Prism software (v.10 GraphPad), with statistical significance set at *p* < 0.05.

For the sensory ratings, repeated measures MANOVA was used to assess differences in mean ratings of the key attributes of the juices with taster status and sex as between-subjects factors, and time as a within-subjects factor. Where differences were identified, ANOVA was used to identify which key attributes showed significant differences for which factors. For RATA data, ANOVA was performed to identify differences that exist for astringency sensations at the two intervention time points. Dietary intake data were analyzed using ANOVA. To check normality, normal quantile (QQ) plots and the Shapiro–Wilk test were used. Spearman’s rank correlation test was performed to evaluate significant correlations between the relative abundance of bacteria and sensory ratings of cranberry and aronia juices in NT and ST subjects. Only bacteria that remained significant after multiple testing corrections were included in the correlation analysis with sensory parameters. The data was analyzed using R software (v.4.3.2) and XLSTAT (v.2023.1). Statistical significance for tests was set at *p* < 0.05.

## 5. Conclusions

This study followed the same 11-day CPE rinse protocol as our previous study [18] but included a larger study population and utilized shotgun metagenomics, which allowed more precise characterization of microbial taxa at the species and strain level. The current study did not replicate our previous finding of baseline differences and intervention effects on the microbial communities of NTs and STs with respect to alpha and beta diversity. Nevertheless, we observed reduced abundance of three bacterial species from Socransky’s classification in the saliva of NTs following the intervention, including *E. corrodens* (green complex), *A. massiliensis* (blue complex), and *C. rectus* (orange complex). All three species may play a role in the development of different features of oral disease. STs showed no decreases in these species but did show an increase in *C. concisus*, which, paradoxically, may be involved in a shift towards higher disease risk. Thus, we conclude that CPE oral rinse had a differential and potentially more positive effect on oral health in NTs.

When all participants were considered, regardless of PROP taster group, we observed an increase in the relative abundance of *S. vestibularis*, which has also been implicated in oral health. Taken together, our findings identify bacterial species that are relevant to the maintenance of oral health and are influenced by CPE delivered as a daily oral rinse. These findings may have important implications for the development of the next generation of oral health products.

It is important to note that different oral sites have different microenvironments in which bacteria are highly adapted to the ecological niche. We collected unstimulated saliva samples, which contain bacteria shed from the tongue, dental plaque, etc., and therefore the profile of bacteria identified in this sample would likely differ from samples collected from other oral sites. The microbial flora in saliva is a unique measure, as the bacteria present are representative of those that colonize other oral sites, hence providing a holistic measurement for the microbes living in the oral cavity. Alternatively, other oral sites like subgingival plaque and tongue samples will contain higher concentrations of bacteria that are representative of biofilm communities, reducing the likelihood that there will be a lack of sufficient DNA to identify bacteria.

Finally, we did not observe differences in our samples for *S. mutans* and *P. gingivalis*, the two most prominent bacteria implicated in dental caries and gingival disease, respectively [83,84]. This is not surprising, since the participants in our study were screened for good oral health and received regular dental care, which might be why the relative abundance of these species was either low or not detected. The next logical step in these studies would be to assess the effects of CPE oral rinse in NTs and STs with and without oral disease.

## Figures and Tables

**Table 1:** Demographic characteristics of the participants.

	Female		Male		Overall
	^1^ NT (*n* = 11)	^2^ ST (*n* = 14)	^1^ NT (*n* = 9)	^2^ ST (*n* = 13)	(*n* = 47)
**Parameter**					
Age, years (mean ± SD)	22.5 ± 5.6	19.9 ± 2.2	19.9 ± 1.5	21.2 ± 2.8	20.9 ± 3.4
BMI, kg/m^2^ (mean ± SD)	23.8 ± 3.7	22.5 ± 3.3	23.6 ± 3.1	23.1 ± 4.5	23.2 ± 3.6
**Race, *n* (%)**					
White	5 (45.5)	2 (14.3)	5 (55.6)	5 (38.5)	17 (36.1)
Hispanic or Latino	1 (9.1)	2 (14.3)	1 (11.1)	2 (15.4)	6 (12.8)
Black or African American	0 (0.0)	2 (14.3)	0 (0.0)	3 (23.1)	5 (10.6)
Asian or Pacific Islander	3 (27.3)	4 (28.6)	3 (33.3)	3 (23.1)	13 (27.7)
Multiracial	2 (18.2)	4 (28.6)	0 (0.0)	0 (0.0)	6 (12.8)
**Highest level of education completed, *n* (%)**					
High School Graduate or GED	1 (9.1)	6 (42.9)	4 (44.4)	2 (15.4)	13 (27.7)
Technical School	0 (0.0)	0 (0.0)	0 (0.0)	1 (7.7)	1 (2.1)
Some College	7 (63.6)	8 (57.1)	3 (33.3)	6 (46.2)	24 (51.1)
College Graduate	1 (9.1)	0 (0.0)	1 (11.1)	3 (23.1)	5 (10.6)
Post Graduate Study	2 (18.2)	0 (0.0)	1 (11.1)	1 (7.7)	4 (8.5)
**Approximate total household income, *n* (%)**					
Less than $5000–$19,999	2 (18.2)	2 (14.3)	1 (11.1)	1 (5.6)	6 (12.8)
$20,000–$49,999	1 (9.1)	1 (7.1)	2 (22.2)	5 (27.8)	9 (19.1)
$50,000–$79,999	4 (36.4)	5 (35.7)	3 (33.3)	11 (61.1)	18 (38.3)
$80,000–Over $100,000	4 (36.4)	6 (42.9)	3 (33.3)	1 (5.6)	14 (29.8)

^1^ NT = non-taster; ^2^ ST = super-taster.

**Table 2:** Baseline logarithmic mean difference (MD) in relative abundance of microbial species in PROP NTs compared to PROP STs.

Phylum	Family	Genus	Species	↑/↓	MD	*p*-Value	Adj. *p*-Value
Bacteroidetes	Prevotellaceae	Prevotella	*Prevotella nigrescens*	**↑**	0.268	0.006	ns
Firmicutes	Lachnospiraceae	Oribacterium	*Oribacterium sinus*	**↑**	0.250	0.036	ns
Firmicutes	Peptiniphilaceae	Peptoniphilus	*Peptinophilus lacrimalis*	**↓**	−0.179	0.016	ns
Firmicutes	Selenomonadaceae	Selenomonas	*Selenomonas _u_s*	**↑**	0.341	0.021	ns
Firmicutes	Selenomonadaceae	Selenomonas	*Selenomonas infelix*	**↑**	0.251	0.016	ns
Proteobacteria	Campylobacteraceae	Campylobacter	*Campylobacter_u_s*	**↑**	0.220	0.043	ns
Proteobacteria	Campylobacteraceae	Campylobacter	*Campylobacter concisus*	**↑**	0.267	0.010	ns
Proteobacteria	Neisseriaceae	Eikenella	*Eikenella corrodens*	**↑**	0.235	0.040	ns
Proteobacteria	Neisseriaceae	Eikenella	*Eikenella halliae*	**↑**	0.236	0.031	ns

The Mann–Whitney U test was used with statistical significance set at *p* < 0.05. *p*-values and adjusted *p*-values using the Holm–Sidak method for multiple comparisons are shown. Non-tasters (*n* = 18), Super-tasters (*n* = 26); Arrows indicate the direction of difference between groups: ↓ = lower in NTs; ↑ = higher in NTs; ns = not significant.

**Table 3:** Baseline versus post-intervention logarithmic mean difference (MD) in relative abundance of microbial species in PROP NTs and PROP STs.

PROP Status	Phylum	Family	Genus	Species	↑/↓	MD	*p*-Value	Adj. *p*-Value
ST	Actinobacteria	Atopobiaceae	Atopobium	*Atopobium_u_s*	**↑**	0.012	0.023	ns
	Actinobacteria	Micrococcaceae	Rothia	*Rothia aeria*	**↓**	−0.171	0.049	ns
	Bacteroidetes	Prevotellaceae	Prevotella	*Prevotella nigrescens*	**↑**	0.106	0.034	ns
	Bacteroidetes	Prevotellaceae	Prevotella	*Prevotella scopos*	**↑**	0.149	0.011	ns
	Firmicutes	Lachnospiraceae	Lachnoanaerobaculum	*Lacnoanaerobaculum orale*	**↑**	0.142	0.012	ns
	Firmicutes	Lachnospiraceae	Lachnospiraceae_u_g	*Lacnospiraceae_u_s*	**↑**	0.157	0.036	ns
	Firmicutes	Streptococcaceae	Streptococcus	*Streptococcus xiaochunlingii*	**↓**	−0.173	0.049	ns
	Firmicutes	Streptococcaceae	Streptococcus	*Streptococcus vestibularis*	**↑**	0.290	0.001	ns
	Firmicutes	Streptococcaceae	Streptococcus	*Streptococcus viridans*	**↓**	−0.039	0.038	ns
	Firmicutes	Veillonellaceaea	Veillonella	*Veillonella_u_s*	**↑**	0.049	0.033	ns
	Proteobacteria	Burkholderiaceae	Lautropia	*Lautropia mirabilis*	**↓**	−0.062	0.043	ns
	Proteobacteria	Campylobacteraceae	Campylobacter	*Campylobacter_u_s*	**↑**	0.169	0.009	ns
	Proteobacteria	Campylobacteraceae	Campylobacter	*Campylobacter consisus*	**↑**	0.180	0.005	ns
	Proteobacteria	Neisseriaceae	Neisseria	*Neisseria polysaccharea*	**↓**	−0.027	0.033	ns
	Proteobacteria	Pateurellaceae	Aggregatibacter	*Aggregatibacter aphrophilus*	**↑**	0.076	0.031	ns
NT	Actinobacteria	Actinomycetaceae	Actinobaculum	*Actinobaculum_u_s*	**↓**	−0.175	0.048	ns
	Actinobacteria	Actinomycetaceae	Actinomyces	*Actinomyces gerencseriae*	**↓**	−0.194	0.022	ns
	Actinobacteria	Actinomycetaceae	Actinomyces	*Actinomyces massiliensis*	**↓**	−0.201	0.007	ns
	Actinobacteria	Corynebacteriaceae	Corynebacterium	*Corynebacterium matruchotii*	**↓**	−0.150	0.016	ns
	Bacteroidetes	Flavobacteriaceae	Capnocytophaga	*Capnocytophaga granulosa*	**↓**	−0.318	0.021	ns
	Bacteroidetes	Flavobacteriaceae	Capnocytophaga	*Capnocytophaga ochracea*	**↓**	−0.294	0.012	ns
	Bacteroidetes	Prevotellaceae	Prevotella	*Prevotella nigrescens*	**↓**	−0.096	0.030	ns
	Bacteroidetes	Prevotellaceae	Prevotella	*Prevotella oris*	**↓**	−0.280	0.005	ns
	Firmicutes	Lachnospiraceae	Oribacterium	*Oribacterium_u_s*	**↓**	−0.192	0.028	ns
	Firmicutes	Lachnospiraceae	Oribacterium	*Oribacterium asaccharolyticum*	**↓**	−0.190	0.014	ns
	Firmicutes	Selenomonadaceae	Selenomonas	*Selenomonas infelix*	**↓**	−0.213	0.001	ns
	Firmicutes	Streptococcaceae	Streptococcus	*Streptococcus sanguinis*	**↓**	−0.117	0.050	ns
	Proteobacteria	Campylobacteraceae	Campylobacter	*Campylobacter rectus*	**↓**	−0.183	0.005	ns
	Proteobacteria	Neisseriaceae	Eikenella	*Eikenella corrodens*	**↓**	−0.273	0.000	0.037
	Proteobacteria	Neisseriaceae	Kingella	*Kingella denitrificans*	**↓**	−0.227	0.014	ns

The Wilcoxon signed-rank test was used to assess mean differences between baseline and post-intervention relative abundance in each group separately, where non-tasters (NT; *n* = 18) and super-tasters (ST; *n* = 26). Statistical significance was set at *p* < 0.05. *p*-values and adjusted *p*-values using the Holm–Sidak method for multiple comparisons are shown. Arrows indicate the direction of change from baseline to post-intervention: ↓ = lower abundance; ↑ = higher abundance; ns = not significant.

**Table 4:** Baseline vs. post-intervention logarithmic mean difference (MD) in relative abundance of selected microbial species in all participants.

Phylum	Family	Genus	Species	↑/↓	MD	*p*-Value	Adj. *p*-Value
Actinobacteria	Atopobiaceae	Atopobium	*Atopobium_u_s*	**↑**	0.075	0.022	ns
Bacteroidetes	Flavobacteriaceae	Capnocytophaga	*Capnocytophaga ochracea*	**↓**	−0.150	0.044	ns
Bacteroidetes	Prevotellaceae	Prevotella	*Prevotella scopos*	**↑**	0.133	0.007	ns
Candidatus Saccharibacteria	Candidatus Saccharibacteria_u_f	Candidatus Saccharibacteria_u_g	*TM7 phylum sp. oral taxon 3*	**↑**	0.045	0.012	ns
Firmicutes	Lachnospiraceae	Lachnoanaerobaculum	*Lacnoanaerobaculum_u_s*	**↑**	0.042	0.040	ns
Firmicutes	Streptococcaceae	Streptococcus	*Streptococcus thermophilus*	**↑**	0.154	0.037	ns
Firmicutes	Streptococcaceae	Streptococcus	*Streptococcus vestibularis*	**↑**	0.233	0.000	0.039
Firmicutes	Veillonellaceae	Veillonella	*Veillonella_u_s*	**↑**	0.033	0.033	ns
Proteobacteria	Burkholderiaceae	Lautropia	*Lautropia mirabilis*	**↓**	−0.089	0.008	ns
Proteobacteria	Burkholderiaceae	Lautropia	*Lautropia dentalis*	**↓**	−0.165	0.044	ns
Proteobacteria	Neisseriaceae	Kingella	*Kingella denitrificans*	**↓**	−0.145	0.032	ns
Proteobacteria	Neisseriaceae	Neisseria	*Neisseria cinerea*	**↓**	−0.132	0.020	ns
Proteobacteria	Neisseriaceae	Neisseria	*Neisseria polysaccharea*	**↓**	−0.065	0.006	ns

The Wilcoxon signed-rank test was used to assess mean differences between baseline and post-intervention in all participants (*n* = 44). The species selected for analysis was based on a cutoff criterion of an increase or decrease of relative abundance in more than 50% of the participants. Statistical significance was set at *p* < 0.05. *p*-values and adjusted *p*-values using the Holm–Sidak method for multiple comparisons are shown. Arrows indicate the direction of change from baseline to post-intervention in logarithmic mean difference in relative abundance: ↓ = lower abundance; ↑ = higher abundance; ns = not significant.

## Data Availability

The data presented in this study are available on request from the corresponding author.

## Supplementary Materials

The following supporting information can be downloaded at: https://www.mdpi.com/article/10.3390/ijms27093935/s1.

## Abbreviations

The following abbreviations are used in this manuscript:

CPE	Cranberry polyphenol extract
PROP	6-n-propylthiouracil
ST	Super-taster
NT	Non-taster
PRP	Proline-rich protein
Cysts	Cystatin
PACs	Proanthocyanidins
CATA	Check-All-That-Apply
RATA	Rate-All-That-Apply
PCA	Principal component analysis
MANOVA	Multivariate analysis of variance
ANOVA	Analysis of variance
DNA	Deoxyribonucleic acid
BMI	Body mass index
MD	Mean difference
NS	Not significant
