# Supplementary material for: Cranberry Polyphenol Extract (CPE) Oral Rinse Improves Salivary Microbiome in 6-n-Propylthiouracil (PROP) Non-Tasters and Palatability of Aronia Juice
Source: Int J Mol Sci. 2026 Apr 28;27(9):3935. doi: 10.3390/ijms27093935 (PMC13163430; doi:10.3390/ijms27093935)
Supplement: Supplementary file 1 [file ijms-27-03935-s001.zip › ijms-4215691-supplementary.pdf]

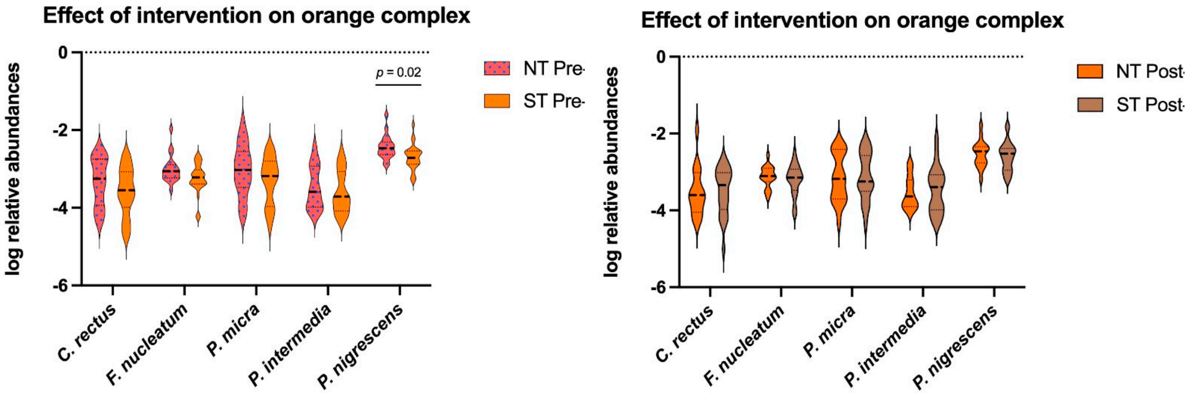

Figure S1. Violin plots showing the log relative abundance of bacteria in Socransky's complexes comparing PROP taster status groups for each intervention time. The orange complex is the only one that showed significant differences and therefore are the only plots shown. Multiple Wilcoxon signed rank test was performed, followed by the Holm-Sidak correction test. Statistical significance is set at $p < 0.05$. NT = non-taster (n = 18), ST = super-taster (n = 26).

Table S1. Reported dietary intakes over the past month (mean ± SEM) stratified by sex and taster status[1,2,3].

		Sex		Taster Status[3]	
**Category**	**Unit**	**Female**	**Male**	**ST**	**NT**
**Weight**	**lbs.**	133.1 ± 4.2[b]	162.5 ± 7.8[a]	145.5 ± 6.7	148.8 ± 6.7
**Protein**	**g**	64.8 ± 6.6	82.7 ± 11.1	73.4 ± 6.2	72.8 ± 13.1
	**% energy**	16.6 ± 0.8	17.4 ± 1.1	17.1 ± 0.8	16.8 ± 1.2
**Carbohydrate**	**g**	180.8 ± 12.8	212.7 ± 14.2	203.2 ± 13.5	184.3 ± 13.3
	**% energy**	49.2 ± 1.4	49.1 ± 2.1	49.2 ± 1.6	49.0 ± 2.0
**Fat**	**g**	57.2 ± 4.8	68.6 ± 7.4	63.5 ± 5.3	60.9 ± 7.5
	**% energy**	34.2 ± 1.0	33.5 ± 1.5	33.6 ± 1.1	34.2 ± 1.4
**Energy**	**kcal**	1482.3 ± 108.0	1805.8 ± 143.4	1678.8 ± 111.6	1565.0 ± 155.1
**Total Sugars**	**g**	68.2 ± 5.3	81.0 ± 7.7	74.8 ± 5.7	73.3 ± 7.9
	**% energy**	4.8 ± 0.3	4.7 ± 0.5	4.7 ± 0.3	4.8 ± 0.5
	**% CHO**	38.9 ± 2.2	37.8 ± 2.4	37.6 ± 1.8	39.4 ± 3.0
**Added Sugars**	**g**	34.7 ± 3.4[b]	49.5 ± 7.0[a]	42.6 ± 4.4	40.1 ± 7.2
	**% energy**	2.6 ± 0.3	3.0 ± 0.5	2.8 ± 0.3	2.7 ± 0.5
	**% CHO**	21.0 ± 2.1	23.6 ± 2.9	22.6 ± 2.0	21.6 ± 3.2
**Dietary Fiber**	**g**	18.5 ± 2.1	19.5 ± 2.9	19.4 ± 2.4	18.4 ± 2.6

[1]Values with different superscript letters are significantly different between sexes by ANOVA at $p < 0.05$. [2]Two subjects were identified as outliers for carbohydrate and sugar intake and were excluded from the analysis (n = 45). [3]NT= non-taster; ST = super-taster.

Table S2. Pre- and post-intervention mean intensity ratings for sensory evaluation of key attributes for cranberry and aronia berry juices stratified by PROP taster status, including ± standard error of the mean (SEM) [1, 3].

	Cranberry Juice				Aronia Berry Juice			
	ST		NT		ST		NT	
**Key Attribute**	**Pre**	**Post**	**Pre**	**Post**	**Pre**	**Post**	**Pre**	**Post**
Sweetness	3.2 ± 0.8	2.9 ± 0.6	3.6 ± 0.6	3.6 ± 0.7	3.4 ± 0.6	4.6 ± 0.6	5.3 ± 0.6	5.7 ± 0.7
Sourness	10.9 ± 0.6	11.4 ± 0.5	10.8 ± 0.6	9.9 ± 0.8	5.5 ± 0.8	3.4 ± 0.5	4.6 ± 0.5	3.6 ± 0.7
Bitterness	6.7 ± 0.9	6.3 ± 0.8	7.3 ± 0.7	6.3 ± 0.9	5.0 ± 0.7	3.3 ± 0.6	4.3 ± 0.7	4.1 ± 0.8
Astringency	7.0 ± 0.8	7.1 ± 0.7	6.8 ± 0.9	7.6 ± 1.0	9.5 ± 0.7	8.7 ± 0.6	10.2 ± 0.8	9.9 ± 0.7
Thickness	3.1 ± 0.5	3.7 ± 0.4	4.3 ± 0.7	4.5 ± 0.8	3.5 ± 0.5	3.8 ± 0.5	4.4 ± 0.6	5.0 ± 0.8
Cranberry/ Aronia Flavor[2]	9.5 ± 0.7	9.3 ± 0.6	9.5 ± 0.6	10.0 ± 0.6	7.0 ± 0.6	7.3 ± 0.6	7.4 ± 0.7	9.2 ± 0.6
Overall Flavor	9.6 ± 0.6	9.6 ± 0.6	9.9 ± 0.6	10.2 ± 0.6	7.4 ± 0.5	7.9 ± 0.6	8.4 ± 0.7	8.2 ± 0.6
Overall Liking	6.9 ± 0.8	7.3 ± 0.7	8.1 ± 0.7	8.6 ± 0.7	6.4 ± 0.5	8.1 ± 0.6	8.0 ± 0.6	8.6 ± 0.6

[1]RM MANOVA was used to detect statistical significance at $p < 0.05$. [2]Cranberry flavor was assessed for cranberry juice samples, while aronia flavor was assessed for aronia berry juice samples. [3]Cells highlighted in green represent an increase from pre-intervention to post-intervention, yellow represents no change, and orange represents a decrease ($n = 47$).

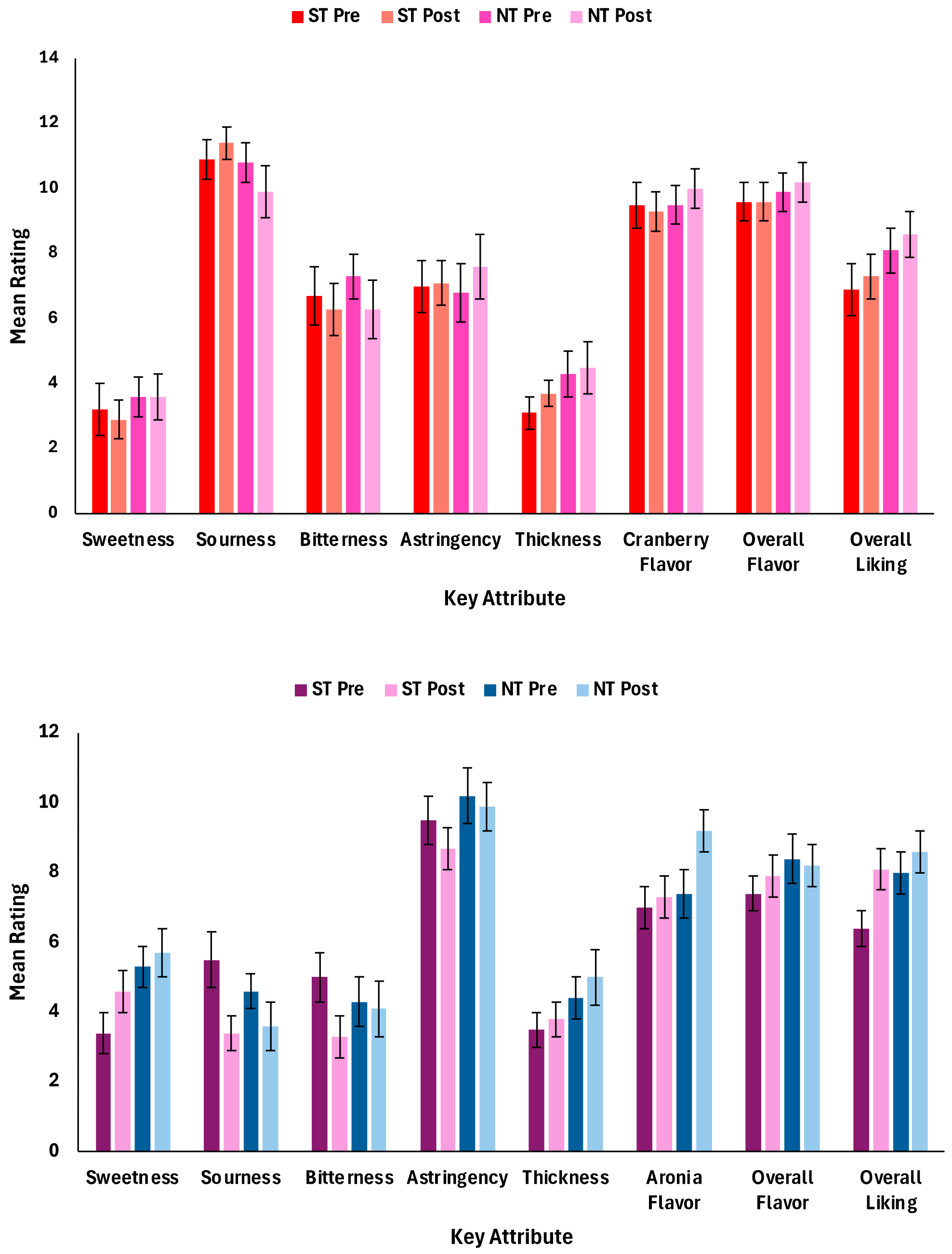

Figure S2. Pre- and post-intervention mean intensity ratings for sensory evaluation of key attributes for cranberry (top) and aronia berry (bottom) juices stratified by PROP taster status. Error bars represent standard error of the mean (SEM). RM MANOVA was used to detect statistical significance at $p < 0.05$. Different letters (a,b) represent significant differences ($n = 47$). The cranberry juice evaluation showed an opposite trend for sourness between the groups - sourness ratings slightly increased for STs at the end of the intervention, while it decreased for NTs. Decreased bitterness and increased overall liking of cranberry juice were unanimous for both groups. For aronia berry juice samples, the decrease in sourness and bitterness perceived, along with the greater overall liking, was largely driven by the STs. These characteristics showed the same trends in NTs, but to a lesser degree. The enhanced aronia flavor was mainly attributed to NTs higher ratings at the end of the intervention. Both groups were found to have increased ratings in sweetness and decreased ratings in astringency.

Table S3. Astringency sensations for cranberry and aronia berry juices at baseline and post intervention for RATA[1].

		Baseline			Post-Intervention		
**Sample**	**Astringency Sensation**	**%**	**n**	**Mean ± SEM**	**%**	**n**	**Mean ± SEM**
Cranberry Juice	Puckering	72	34	8.01 ± 0.6	70	33	8.53 ± 0.7
	Roughing	27	12	5.87 ± 1.1	38	17	5.75 ± 0.9
	Drying	73	33	6.04 ± 0.6	64	29	5.93 ± 0.7
Aronia Berry Juice	Puckering	49	23	8.37 ± 0.9	49	23	6.52 ± 0.6
	Roughing	67	31	9.55 ± 0.6	67	31	8.5 ± 0.6
	Drying	94	44	9.67 ± 0.5	94	44	8.67 ± 0.6

[1]Shows the percentage and number of subjects, along with mean ± standard error of the mean (SEM) for intensity ratings (n = 47). Overall, aronia berry juice had higher baseline ratings. Most participants reported experiencing drying and puckering for cranberry juice, while the majority reported experiencing drying and roughing for aronia berry juice. For cranberry juice, there was an increasing trend for puckering, while for aronia berry juice, there was a decreasing trend for all astringency sensations.
